# Cytoplasmic Antineutrophil Cytoplasmic Antibodies (C-ANCA) Vasculitis: An Uncommon Complication After Stem Cell Transplantation

**DOI:** 10.7759/cureus.25445

**Published:** 2022-05-29

**Authors:** Ahmad Raja, Summia Matin Afridi, Myint M Noe, Akriti Jain

**Affiliations:** 1 Internal Medicine, Mary Imogene Bassett Hospital, Cooperstown, USA; 2 Infectious Disease, Lee Health, Fort Myers, USA; 3 Internal Medicine, AdventHealth East Orlando, Orlando, USA

**Keywords:** vasculitis, internal medicine and rheumatology, transplant recipient, granulomatosis with polyangiitis (gpa), auto immune

## Abstract

Granulomatosis with polyangiitis (GPA) is a rare, autoimmune, antineutrophil cytoplasmic antibody (ANCA)-associated vasculitis of uncertain etiology. The incidence of autoimmune complications following stem cell transplant is around 2-5%, with autoimmune cytopenia reported most frequently. We present a case of a 65-year-old male patient who presented to the hospital with productive cough, dyspnea, and fever for five months after haploidentical stem cell transplantation. On presentation, he was febrile, tachypneic, and mildly hypoxic. Chest radiograph showed bilateral pulmonary infiltrates. An initial diagnosis of pneumonia was made, and the patient was started on antibiotics. The patient did not respond to initial management, and all his initial infectious workups came back negative. On further evaluation, cytoplasmic antineutrophil cytoplasmic antibodies (c-ANCA) resulted positive in high titers. The patient was diagnosed with GPA, and IV methylprednisolone and rituximab were started. He responded well to treatment and was eventually discharged home. The classical form of GPA is characterized by the involvement of the upper respiratory tract, sinuses, lungs, and kidneys. Autoimmune disorders may develop secondary to hematopoietic stem cell transplant (HSCT). In our case, the patient was diagnosed with GPA, which is likely one of the autoimmune complications after HSCT.

## Introduction

Granulomatosis with polyangiitis (GPA) is a rare, autoimmune, antineutrophil cytoplasmic antibody (ANCA)-associated vasculitis of uncertain etiology. It is much more prevalent among whites and most commonly affects the older population, but cases have been reported in all age groups [1-3]. This disorder is characterized by necrotizing and granulomatous inflammation of small vessels [4,5]. Hematopoietic stem cell transplant (HSCT) is one of the treatment options for several malignant and non-malignant diseases, including hemoglobinopathies and autoimmune and immunodeficiency disorders [6]. Several autoimmune disorders have been reported to occur following HSCT. The underlying pathogenetic mechanisms for the development of autoimmunity are hypothesized to involve the impaired function of regulatory T cells leading to their unchecked activation [7,8]. The incidence of autoimmune complications following stem cell transplant is around 2-5%, with autoimmune cytopenia reported most frequently [6,9]. We report a case of GPA vasculitis presenting with pulmonary symptoms five months after a successful stem cell transplant.

## Case presentation

**Figure 1:**
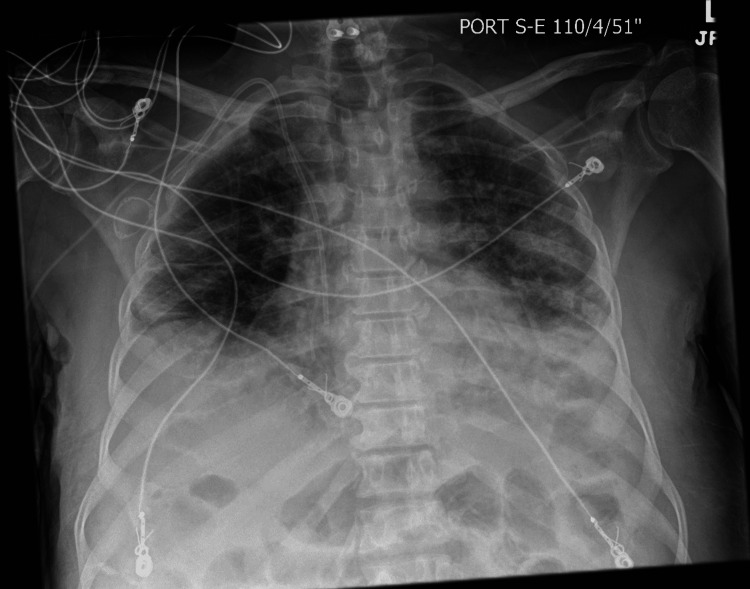
Chest X-ray showing bilateral pulmonary infiltrates.

**Figure 2:**
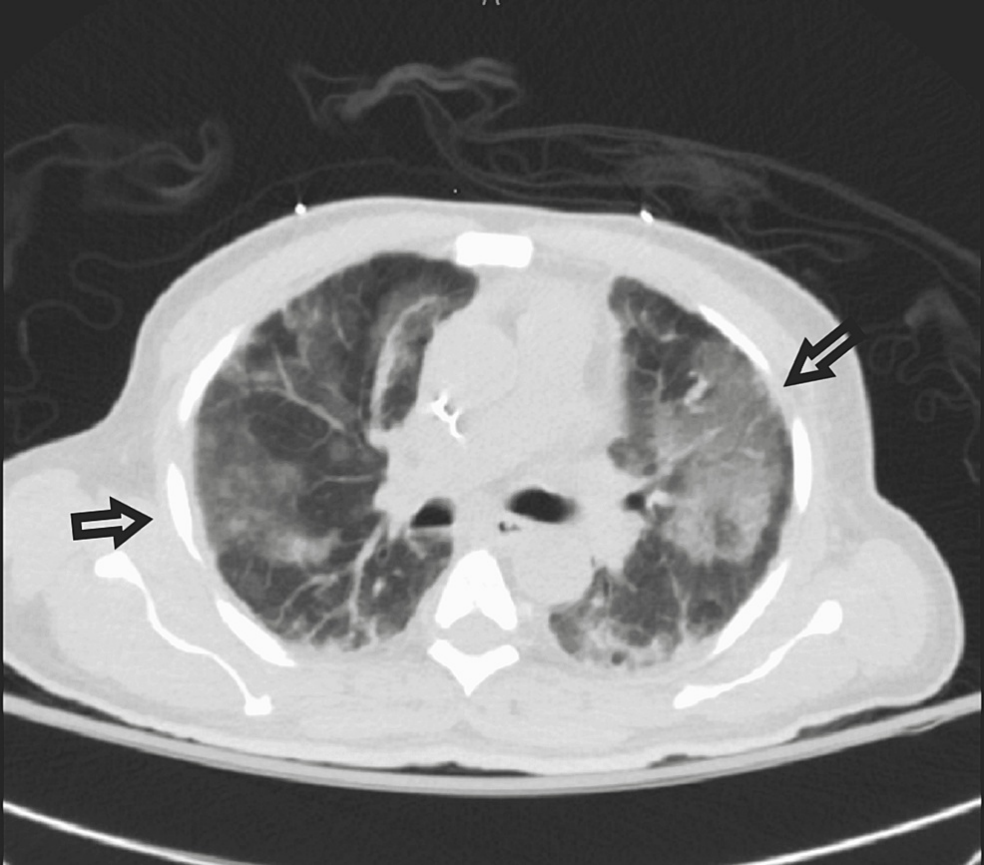
CT chest showing bilateral multiple pulmonary opacities (arrows).

A 65-year-old male underwent haploidentical stem cell transplantation after the diagnosis of myelodysplastic syndrome (MDS). The medical history of the donor is unknown. The patient did not have any previous medical history of autoimmune diseases, including vasculitides. His post-transplant condition remained well until five months later, when he presented with a two-week history of a productive cough, fever, and dyspnea. He was admitted for presumptive bacterial pneumonia. On admission, he was febrile (temperature 100.5 F), tachypneic (respiratory rate 22 breaths per minute), had a blood pressure of 117/75 and had an oxygen saturation of 88% on room air. Chest auscultation revealed bilateral crackles in the lower lung fields. Labs were consistent with leukocytosis (WBC count 13 x103 cells/uL), normochromic normocytic anemia (hemoglobin 10g/dL), normal platelet count, and a normal creatinine of 0.92 mg/dL. His electrolytes, liver function tests, and urinalysis were normal. Chest radiograph showed bilateral pulmonary infiltrates (Figure 1). The patient was placed on 2 liters of oxygen by nasal cannula and started on empiric IV broad-spectrum antibiotics (meropenem and vancomycin). CT scan of his chest showed bilateral multiple pulmonary opacities (Figure 2).

The patient’s respiratory status deteriorated two days later with increasing oxygen requirements and worsening respiratory distress, for which he required endotracheal intubation and mechanical ventilation. Transbronchial bronchoscopy was performed, which showed normal lung parenchyma. Bronchoalveolar lavage fluid was significant for diffuse alveolar hemorrhage but negative for cultures, polymerase chain reaction (PCR), and stains for viruses, bacteria, pneumocystis, and fungi. On further autoimmune workup, cytoplasmic antineutrophil cytoplasmic antibodies (c-ANCA) came back positive (>8 U). In addition, anti-nuclear antibody (ANA), rheumatoid factor (RF), anti-Ro antibody, and anti-La antibody also came back negative. The patient was started on IV methylprednisolone 2mg/kg every eight hours, and over a few days, the patient’s clinical condition improved. Based on the clinical presentation, radiological and lab data, a diagnosis of GPA was made, and rituximab was added to the treatment regimen. Cyclophosphamide was not considered because of the risk of myelosuppression in this patient with an MDS history. He responded well to the treatment and was weaned off mechanical ventilation successfully. He was eventually discharged home on oral steroid taper and follow-up with rheumatology to continue Rituximab therapy.

## Discussion

The classical form of GPA is characterized by the involvement of the upper respiratory tract, sinuses, lungs, and kidneys. The hallmarks of this condition are systemic necrotizing vasculitis, necrotizing granulomatous inflammation, and necrotizing glomerulonephritis [10]. In the limited form of the disease, the kidneys are usually spared. It is well-known that C-ANCA/PR3-ANCA is highly specific for GPA. The diagnosis is made based on clinical manifestations of vasculitis and histological evidence of granulomatous inflammation or necrotizing vasculitis [10]. Autoimmune disorders may develop secondary to HSCT, with autoimmune cytopenia and autoimmune thyroid disease being the most common [11]. Other documented autoimmune disorders include myasthenia gravis, immune-mediated neuropathies, rheumatoid arthritis, and Lupus-like illness. There is also a reported case of cerebral vasculitis in a patient with non-Hodgkin lymphoma and bone marrow transplant [12]. Cases have been reported about c-ANCA vasculitis with pulmonary and renal involvement; however, in our case, the patient did not have kidney disease [13]. HSCT has a significant effect on the immune system. In the early phases, the transplant lymphocytes amplify in the presence of growth factors, called homeostatic expansion. Later, the recipient’s lymphocytes with a different set of receptors transpire. This starts a cascade of events that eventually lead to the formation of uncontrolled autoreactive T-cells, triggering autoimmunity [14]. It has also been reported that the underlying pathophysiology of chronic graft-versus-host disease (GVHD) is similar to autoimmune diseases like scleroderma [6]. GVHD remains in the differential, but the sites of involvement are usually the GI tract, liver, and skin, with lungs being less commonly involved [15]. The diagnosis of GPA can be challenging. A tissue biopsy can be obtained depending on the site of involvement, for example, skin, nasal, or kidney. A surgical biopsy of the lung tissue can be useful if there is lung involvement, but this procedure is associated with a high risk of complications [16]. In our case, the patient was diagnosed with GPA, which is likely one of the autoimmune complications of HSCT, given the recent history of transplantation in the absence of other identifiable triggers. The diagnosis of GPA was made based on the patient’s clinical history, positive serologic antibody test, and bilateral pulmonary opacities seen on CT. A lung biopsy was not performed due to the patient’s clinical status and high risk of morbidity. GVHD was unlikely given the lack of GI tract, liver, or skin involvement and positive c-ANCA antibody. Infections are potential triggers for post-transplant vasculitis; however, no infection was documented in our case.

## Conclusions

In summary, our case report suggests that ANCA-associated vasculitis may develop in the setting of stem cell transplantation and must be recognized promptly in the correct clinical setting since it has a high morbidity and mortality rate if left untreated.
